# Super-selective Embolisation and Surgical Excision of the Facial Arteriovenous Malformation

**DOI:** 10.7759/cureus.57240

**Published:** 2024-03-30

**Authors:** Ramsundar Chaulagain, Ashi Chug, Saurabh Simre, Sameer Pandey, Sudarshan Shrestha

**Affiliations:** 1 Oral and Maxillofacial Surgery, All India Institute of Medical Sciences, Rishikesh, Rishikesh, IND

**Keywords:** resection, intraoral approach, superselective embolisation, face, arterivenous malformation

## Abstract

Vascular anomalies broadly include vascular tumours and malformations. Arteriovenous malformations (AVM), though rare in the oral and maxillofacial regions, can present with swelling, facial asymmetry, ulceration, and bleeding tendencies, which can be life-threatening. Thus, to minimise the associated life-threatening consequences, prompt and appropriate diagnosis of the lesion is necessitated. The management of the AVM is a therapeutic challenge for maxillofacial surgeons; however, technological advances in interventional radiology have gained a foothold. Super-selective embolisation of the feeder vessels with subsequent resection of the lesion is the most widely accepted approach for management. The present report describes a unique case of a facial AVM managed through a trans-oral approach without any post-operative sequelae.

## Introduction

Vascular lesions, traditionally known as vascular anomalies, are a heterogeneous group of congenital blood vessel disorders [1]. The International Society for the Study of Vascular Anomalies (ISSVA) has subcategorized it into vascular tumours and malformations, each characterised and differentiated based on specific morphology, clinical features, pathophysiology, and treatment approach [2]. Arteriovenous malformation (AVM) is the anomalous vascular channel between the artery and vein formed by bypassing the capillary bed [3]. Vascular tumours are primarily congenital lesions commonly occurring in children that show increased mitotic activity leading to cell proliferation. AVM, on the contrary, has normal cells and occurs due to a morphogenic defect [4]. They can be arterial, venous, lymphatic, AVM, or arterio-venous (AV) fistulae [5].

Based on fluid dynamics, vascular malformations can be high-flow or low-flow lesions [5]. AVM are rare high-flow lesions, comprising only 1.5% of vascular anomalies and 50% presenting in the maxillofacial region [6]. Management of AVM is challenging since it poses a significant risk of bleeding and related complications. Various therapeutic options, such as sclerotherapy, embolisation, laser, surgical excision, and combinations, are mentioned in the literature [4,7-9]. However, owing to the complications associated with and recurrence of the lesion, a proper diagnosis and treatment plan should be formulated to avoid related morbidity and mortality. The case report describes the AVM of buccal mucosa, which was managed with pre-operative super-selective embolisation of feeder vessels and subsequent surgical excision.

## Case presentation

A systemically healthy male in his 20s reported to the department with a complaint of swelling over the right cheek two months ago. The patient reported acute, rapidly progressive swelling, sudden in onset over the right cheek region, associated with mild to moderate, dull aching, and non-radiating pain. There was no reported history of bleeding or discharge. The patient received antibiotics for a few days after consulting the local practitioner. The swelling gradually decreased in size after medication.

**Figure 1:**
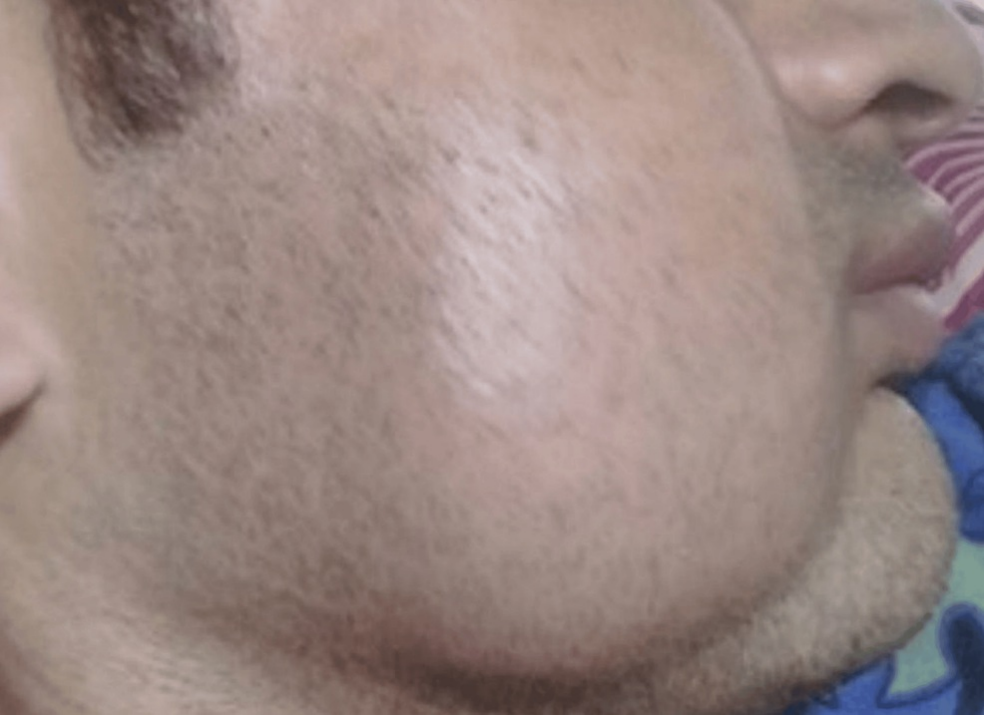
Initial clinical presentation of the patient with rapidly progressive swelling on the right cheek

Extra-oral examination revealed a single, well-defined, soft, non-tender ovoid swelling of size 8 cm × 6 cm over the right cheek region (Figure 1).

The swelling was non-compressible and pulsatile, and the overlying skin and intraoral mucosa were unremarkable. No surface ulceration or bleeding was appreciated.

**Figure 2:**
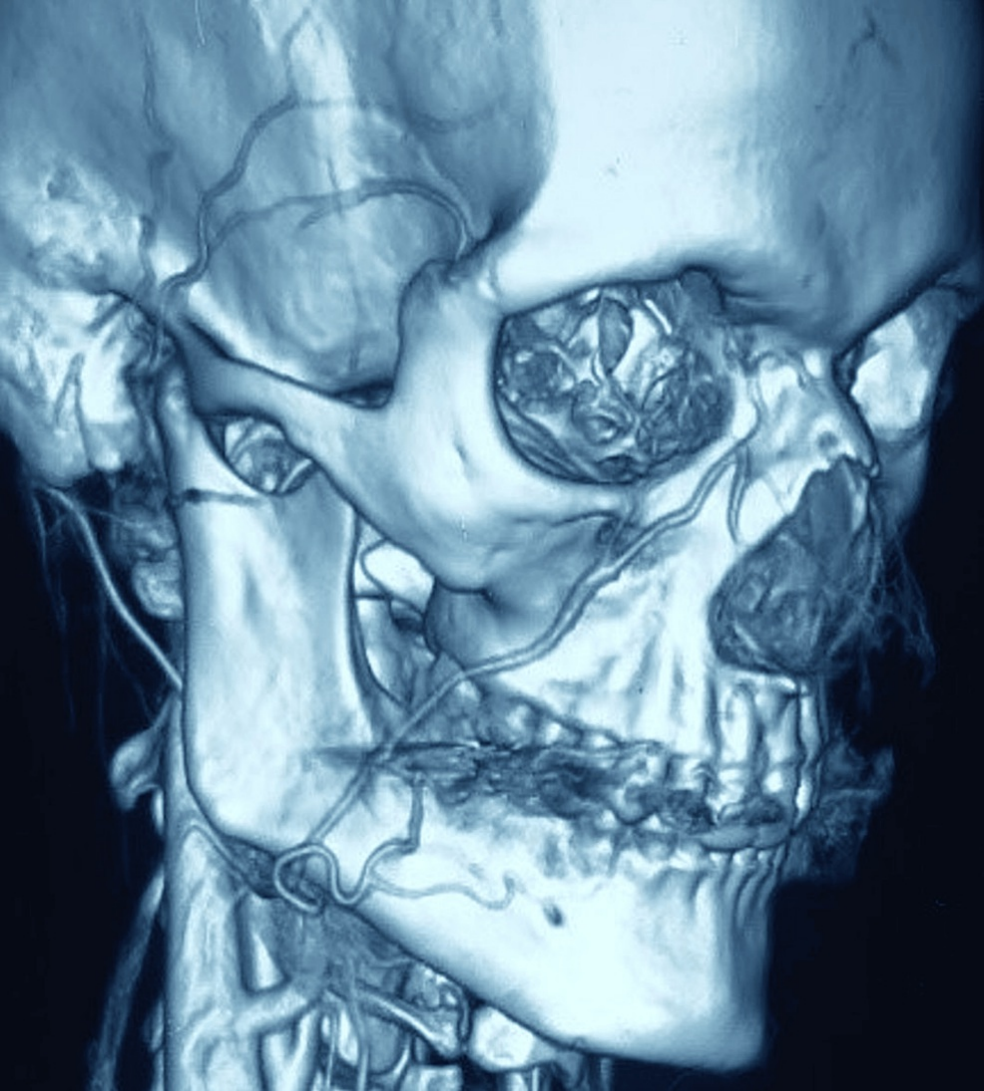
3D reconstruction of the CT angiography showing the abnormal branching from the right facial artery at the body of mandible

**Figure 3:**
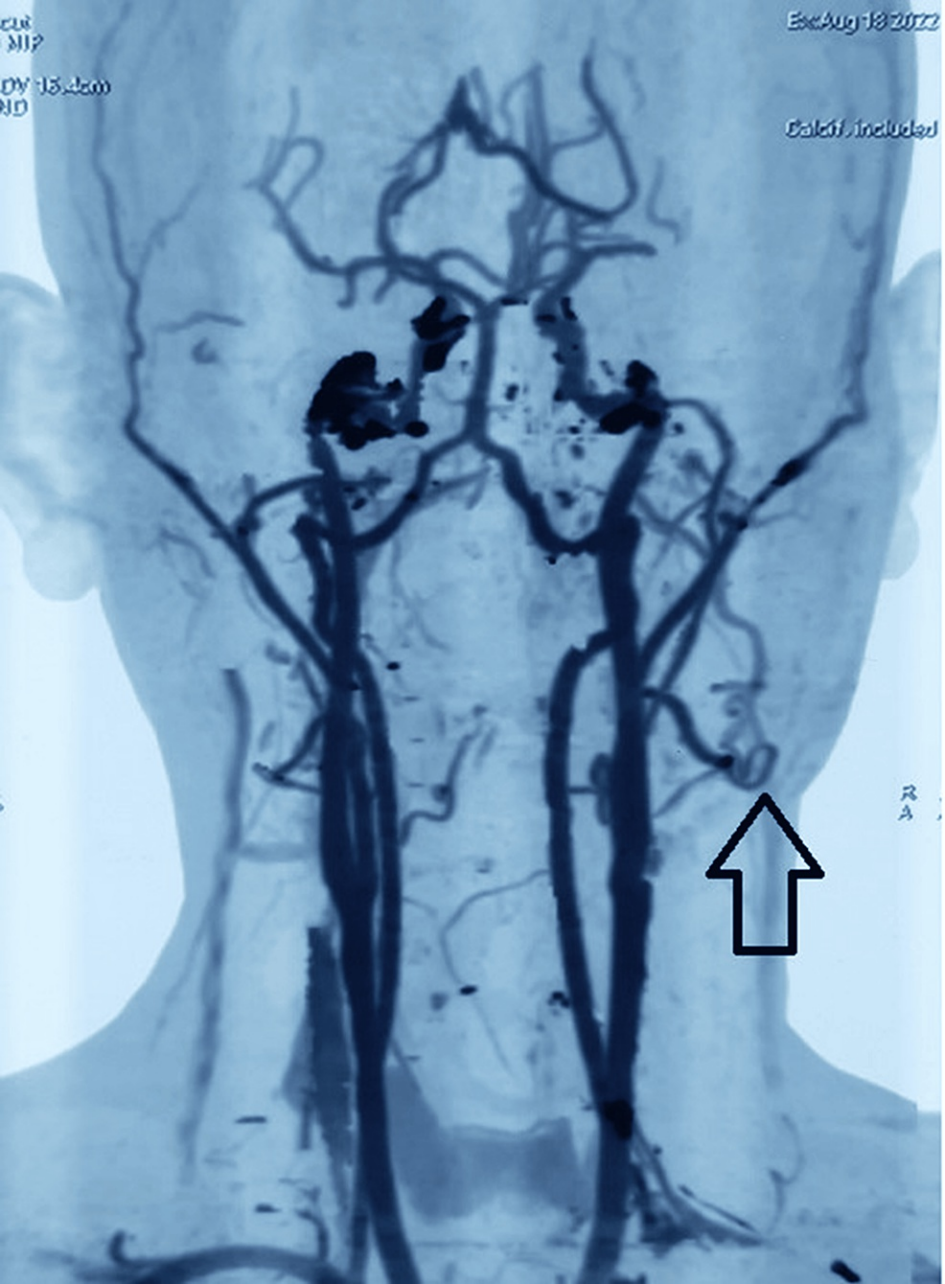
Trans-arterial catheter angiography for depicting the arterio-venous shunting of right facial artery and vein

A colour Doppler ultrasound of the right cheek was opined, which revealed an ill-defined soft tissue lesion of 4.2 cm × 1.3 cm in the subcutaneous plane, showing multiple anechoic areas without internal vascularity, suggestive of a low-flow lymphovascular lesion or involuting hemangioma. Thus, with the suspicion of a likely vascular anomaly, computed tomography (CT) angiography of the face was advised. CT angiography revealed the hypo-dense enhancing lesion in the subcutaneous issue in the right hemi-mandible with arterial supply from the facial artery and draining to the facial vein (Figures 2-3).

The initial diagnosis of the vascular lesion was established based on the clinical presentation; however, the dilemma of the type of lesion persisted. Capillary malformation was distinguished since the extra-oral skin and buccal mucosa were unremarkable, had no supportive medical history, and had an acute presentation. In contrast to infantile hemangiomas, the lesion did not exhibit a tumour-like solid appearance, which was reflected in the imaging as well. The lack of clear circumscription, the absence of lesions in childhood, and the lack of proportional growth of lesions demarcated from the congenital hemangiomas. The traumatic arteriovenous fistulae can also progress to AVM; however, the medical history is non-indicative. CT angiography revealed the supply from the facial artery and the draining of the facial vein, confirming the diagnosis. AVM-related syndromes should be considered and distinguished from simple arteriovenous malformations.

**Figure 4:**
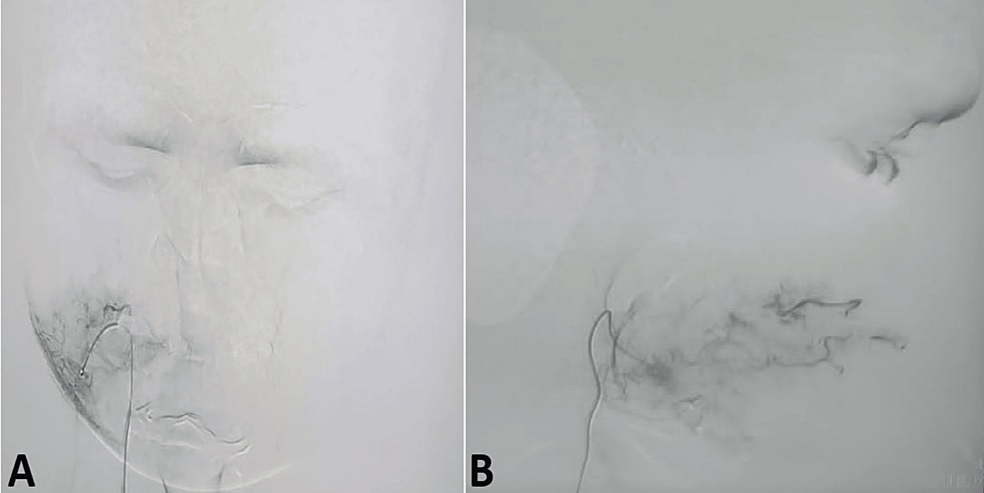
Digital subtraction angiography images (A) Frontal view and (B) lateral view

The patient was planned for preoperative super-selective embolisation of the feeder vessels supplying the lesion. The external carotid artery was hooked, and an angiogram revealed a right cheek abnormal blush (Figure 4).

**Figure 5:**
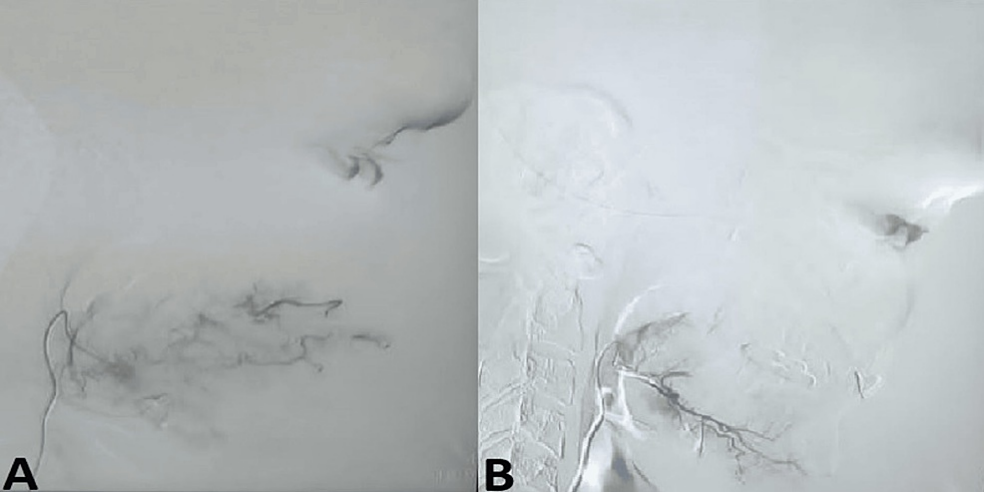
Digital subtraction angiography images (A) Pre-embolization and (B) post-embolization

The feeder artery and the branch of the right facial artery were selectively hooked with a micro-catheter, and further angiograms demonstrated vascular malformation. Embolisation was performed with polyvinyl chips (PVC) particles (300-500 µm) of the branch of the right facial artery and collateral vessels. A post-embolisation angiogram showed loss of abnormal blush in the right cheek, confirming adequate embolisation (Figure 5).

**Figure 6:**
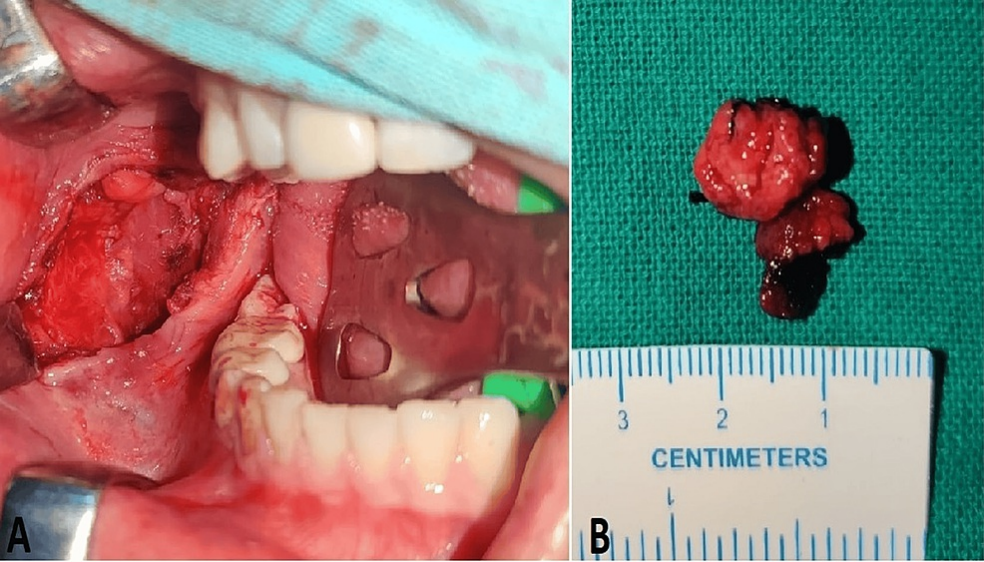
Intraoperative image (A) and excised specimen (B)

A complete surgical excision of the AVM was done 48 hours post-embolisation under general anaesthesia through a trans-oral approach without any uneventful complications (Figure 6).

**Figure 7:**
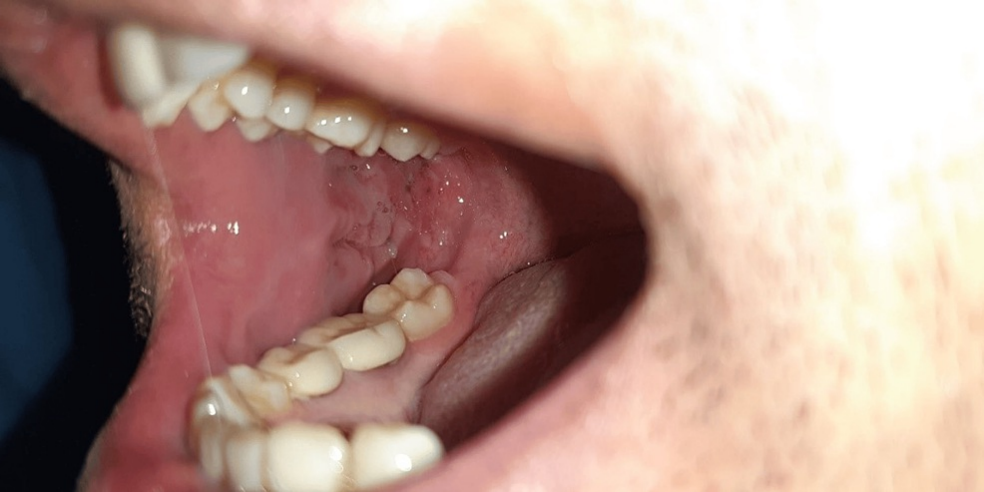
Follow-up image after six months with surgical site healed completely and no recurrence seen

The patient was discharged after two days of hospital stay. The histopathological study of the excised lesion showed features consistent with the AVM. The surgical site healed uneventfully, with no recurrence reported after six months of follow-up (Figure 7).

The young patient in his twenties of life resumed his duty following the surgery.

## Discussion

Historically, AVM was described as snakes covering the Greek god Gordon’s head [7]. AVM, though rare, is a progressive and persistent lesion that can be life-threatening. The exact mechanism of the development of the AVM is still unknown; however, the exacerbation of the symptoms and growth of the lesion can be attributed to the trauma and post-thrombotic ischemic changes, and a positive correlation is seen with the hormonal changes during puberty [4]. In the maxillofacial region, the lesion is common in the tongue, lip, palate, gingiva, and buccal mucosa. Kohuta et al., in their 81 cases of AVM occurring in the head and neck region, reported the incidence in the cheek (31%), ear (16%), 10% each in the forehead and nose, upper lip (7%), mandible, and neck, respectively, and 5% and 4% incidence in the scalp and maxilla [10]. The manifestation can vary based on the location, extent, and size of the lesion. Extra-orally, the presentation can be simple hue or discoloration, facial asymmetry, swelling with palpable thrills or bruises, ulceration, haemorrhage, and bleeding. In this case, the swelling was pulsatile and sudden in onset [6,10]. Intraoral lesions can impair mastication, speech, and swallowing. AVM, when present intraosseously, can cause gingival bleeding, mobility of teeth, and occlusal discrepancy. Furthermore, such lesions lead to significant bleeding following the extraction, which can be life-threatening [10]. Various syndromes, such as Bonnet-Dechame-Blanc syndrome, capillary malformation-AVM syndrome, Parkes-Weber syndrome, and Cobb syndrome, are known to be associated with AVM [11].

Various imaging modalities, such as colour Doppler ultrasonography, contrast-enhanced computed tomography (CECT), magnetic resonance imaging (MRI), CT angiography, and digital subtraction angiography (DSA), can be used for diagnosis and the therapeutic purpose of AVM. However, the choice of investigation depends upon the nature of the lesion, the merits and demerits of the imaging technique, and the available resources. USGs are useful diagnostic aids in AVM, but their limited tissue penetrability cannot detect deeper and intra-bony lesions, despite being the least invasive and cheaper technique [8,12]. Colour Doppler ultrasonography helps to identify the flowing nature of the lesion. CT scans can be a choice of investigation for intra-bony lesions. The intraosseous lesions can be appreciated for their ill-defined radiolucency or honeycomb/soap bubble appearance, which cannot be diagnostic. CECT images identify AVM as highly enhancing lesions and depict soft tissue enhancement as well as the dilated feeder and the draining vessel. MRI is the generally preferred investigation in suspected AVM cases since plain radiographs and computed tomography have limited roles [12]. It shows a good depiction of the vascular structure along with the differentiation of the flow. DSA is applicable for identifying the feeding vessel, collateral vessel, and draining vessel, as well as therapeutic endovascular intervention procedures like embolisation under intervention radiology [8]. Histopathological findings of AVM include focal areas of haemorrhage with communicating vascular channels of variable size lined with endothelial tissue. Secondary features like atherosclerosis, thrombosis, and calcification may also be evident. Dilated and torturous lymphatic channels can be seen in cases of lymphatic malformations [7].

AVM poses a therapeutic challenge for surgical management because of its hemodynamic characteristics. Active intervention measures are used in large lesions with facial asymmetry, impeding functional activities like swallowing, speech airway, ulcerations, bleeding, and high-flow lesions, which may produce cardiac decompensation [10]. The literature describes the different treatment strategies, which can be broadly divided into (i) interventional therapy and (ii) surgical excision.

Interventional therapy includes minimally invasive percutaneous sclerotherapy and embolisation procedures [12]. Various sclerosing agents, such as absolute alcohol, pingyangmycin (PYM), OK-432 (picibanil), polidocanol, ethanolamine oleate, bleomycin, doxycycline, and sodium tetradecyl sulphate, have applications in vascular malformation management. Embolising agents such as ethanol, cyanoacrylate, and polyvinyl particles obliterate the nidus, arteriovenous connections, and dilated venous system. Embolisation obstructs the feeder vessels, reduces the lesion size, and prevents hemorrhagic episodes. However, embolisation as the only method of management can be unsuccessful due to the growth of the collateral blood vessel and neovascularization [8].

Surgical excision of the lesion can be carried out safely in low-flow lesions, but an assessment for possible coagulopathy and recurrences is indicated. Ligation of the feeder vessel is not a choice since it complicates any further interventional procedure in the future in cases of recurrence. Pre-surgical embolisation obliterating the feeder vessel helps to reduce intraoperative bleeding and associated potentially life-threatening complications. Thus, embolisation, along with surgical excision, is the most widely accepted approach for the management of AVM [9]. The extra-oral approach has been preferentially used for the surgical excision of the lesion since the surgeons can have good control over the vasculature. However, in this case, we underwent the preoperative embolisation 48 hours prior, and successful excision of the lesion was done via a trans-oral approach without any complications. Careful dissection and surgical judgement were key to the successful management of the lesion, which helped address the patient's apprehension regarding the aesthetic outcome of the extra-oral approach. Hence, clinical suspicion with appropriate investigation modalities for diagnosis along with multimodal management facilitates avoiding morbidity and mortality concerning AVM [6].

## Conclusions

Clinical suspicion with the appropriate mode of investigation helps in prompt diagnosis, avoiding morbidity related to the vascular lesions. Various modalities of management have been described in the literature; however, the treatment modality is guided by the location, size, and extent of the lesion, along with the patient’s concern. Pre-operative embolisation followed by surgical excision is the most accepted modality of management in cases of AVM. Regular follow-up and evaluation are essential to rule out the success of the treatment and the incidence of recurrence.
